# Distance Estimation Is Influenced by Encoding Conditions

**DOI:** 10.1371/journal.pone.0009918

**Published:** 2010-03-29

**Authors:** Anna Oleksiak, Mirosława Mańko, Albert Postma, Ineke J. M. van der Ham, Albert V. van den Berg, Richard J. A. van Wezel

**Affiliations:** 1 Utrecht Institute for Pharmaceutical Sciences, Utrecht University, Utrecht, The Netherlands; 2 Functional Neurobiology and Helmholtz Institute, Utrecht University, Utrecht, The Netherlands; 3 Experimental Psychology and Helmholtz Institute, Utrecht University, Utrecht, The Netherlands; 4 Biomedical Signals and Systems Group, University of Twente, Enschede, The Netherlands; University of Minnesota, United States of America

## Abstract

**Background:**

It is well established that foveating a behaviorally relevant part of the visual field improves localization performance as compared to the situation where the gaze is directed elsewhere. Reduced localization performance in the peripheral encoding conditions has been attributed to an eccentricity-dependent increase in positional uncertainty. It is not known, however, whether and how the foveal and peripheral encoding conditions can influence spatial interval estimation. In this study we compare observers' estimates of a distance between two co-planar dots in the condition where they foveate the two sample dots and where they fixate a central dot while viewing the sample dots peripherally.

**Methodology/Principal Findings:**

Observers were required to reproduce, after a short delay, a distance between two sample dots based on a stationary reference dot and a movable mouse pointer. When both sample dots are foveated, we find that the distance estimation error is small but consistently increases with the dots-separation size. In comparison, distance judgment in peripheral encoding condition is significantly overestimated for smaller separations and becomes similar to the performance in foveal trials for distances from 10 to 16 degrees.

**Conclusions/Significance:**

Although we find improved accuracy of distance estimation in the foveal condition, the fact that the difference is related to the reduction of the estimation bias present in the peripheral conditon, challenges the simple account of reducing the eccentricity-dependent positional uncertainty. Contrary to this, we present evidence for an explanation in terms of neuronal populations activated by the two sample dots and their inhibitory interactions under different visual encoding conditions. We support our claims with simulations that take into account receptive fields size differences between the two encoding conditions.

## Introduction

Visual information about object locations in the nearby environment is acquired either by bringing these items onto the fovea with an eye movement or by encoding their presence by peripheral vision. While the foveal visual field most often samples information important for the current behavior, peripheral vision allows us to locate other potentially interesting objects that could become the target of the next saccade. It has been shown that positional uncertainty, substantiated in a higher variability and error of localization performance, increases with eccentricity [1]–[5]. If one takes this fact into account, a straightforward prediction follows that encoding of an object position will be more accurate when an observer fixates that object than when his/her gaze is focused elsewhere in the visual field.

While there is a plethora of research that tested the upper limit of relative spatial localization performance [6]–[9], it is still unknown how object positions relative to one another at a larger scale are encoded (cf. [10]). This question is complicated by the fact that while fixating one item, the other necessarily is encoded peripherally. The two encoding conditions may result in different estimation biases. Furthermore, object positions are perceived closer to the fovea than in reality and this ‘foveal attraction’ effect can be exaggerated by a working memory component of the task [11]–[13]. Yet another type of bias can be expected to emerge in distance estimation performance, a so-called repulsion effect observed e.g., in motion direction perception [14], [15], orientation discrimination [16]–[19], and stereoscopic depth perception [20], [21]. This effect is instantiated in perceiving compared orientations or motion directions as been more distinct than they actually are. Importantly, the range of occurrence of this effect depends on the neurons tuning curve width, which directly translates into the size of the neurons' receptive fields (RF). That is, the larger the RFs of the population of neurons influencing the percept, the greater the range of the feature values that would yield the repulsion effect. If spatial interval perception were influenced by the repulsion effect, one would anticipate differences in its size and range between peripheral vision that is dominated by the large RFs and the foveal vision that samples information through small RFs.

Taking all these scenarios under consideration, foveal encoding of locations delimiting a spatial interval might differ from peripheral conditions in at least three measures of performance: general accuracy of estimates (absolute error), variability of responses (scatter of estimates) and estimation biases (signed errors). Here we assess these likely differences of estimates of a distance between the two visual encoding conditions: foveal and peripheral. The volunteers memorized and after a brief delay reproduced with a mouse pointer the distance separating two discrete dots in the frontoparallel plane (2D). By placing the movable cursor dot relative to a stationary reference dot subjects could indicate the memorized spatial interval along the horizontal dimension.

In agreement with previous reports [4]–[13], [22]–[26] we observed an improved accuracy of spatial judgments in the condition where the observers could foveate the sample dots. Importantly however, such effect was observed only for smaller distances and the improvement actually reflected a reduction in distance overestimation bias apparent in the peripheral condition. These aspects challenge the notion that foveal encoding decreased the eccentricity-related positional accuracy in comparison with peripheral encoding. Contrary to that, we favour the ‘repulsion effect’ explanation that takes into account inherent differences between foveal and peripheral encoding of spatial information which correlate with differences in average receptive field sizes involved in visual encoding. We support this notion with model simulations that take into account the RFs sizes inherent to the two encoding conditions.

## Methods

### Participants and ethics

Nine human observers with normal or corrected-to-normal vision took part in the experimental sessions but data from two persons were removed due to a low percentage (less than 50%) of trials with good eye tracker signals and conforming to the instructions. One of the subjects was the author (A.O.), whereas the remaining persons were unaware of the exact hypothesis and predictions behind the study. They were, however, informed that the purpose of the study is to measure the accuracy of distance estimates in different visual conditions and that the eye movement recordings served as a check for the compliance with instructions. This explanation was followed by a demonstration of stimuli and instructions, after which the volunteers gave consent to participate in the experimental sessions for a monetary reward. The experiment was conducted in accordance with Utrecht University ethics and safety guidelines, however, we did not feel that ethics approval was necessary for this study.

Each of the observers completed either eight or ten 10-minute sessions depending on their eye position data (frequency of blinks, significant head movements and number of trials where the instructions were confused). For two volunteers the gaze position recordings revealed a very high frequency of blinks or relatively large head movements and/or deviation from the instructions. Consequently, the percentage of trials where we could gather reliable eye position coordinates was below 50% so we removed the data from these two volunteers from the analyses reported here. The pattern of results and conclusions, however, were not affected by exclusion of these two subjects.

### Apparatus

The experiment was written in Matlab (version R2007b), with the aid of the Psychophysics and Eyelink Toolbox extensions [27]–[29]. Stimuli were presented on a 20-inch COMPAQ monitor with a resolution of 1024×768 pixels and a monitor refresh rate of 100 Hz. Participants were seated 65 cm from the monitor inside a darkened room with a bite-board in their mouth that prevented them from making any significant head movements.

To get an indication of how well the subjects followed the instructions their gaze-position was monitored with a video-based tracker (Eyelink® II version 2.02, SR Research, Mississauga, Ontario, Canada) in a pupil only mode at a sampling rate of 500 Hz and average accuracy of less than 0.5 deg. Though, viewing was binocular, only the left eye was tracked. The gaze position data was parsed online with a saccadic threshold of 22 deg per second, which allowed detection of saccades as small as 0.3 deg. Before each of the 10-minute sessions the apparatus was calibrated by having the observer fixating a single dot successively appearing at nine different positions on the monitor. In the course of each session drift correction was performed manually by the experimenter monitoring the eye tracker display using as a reference fixation period before sample onset.

### Stimuli

**Figure 1:**
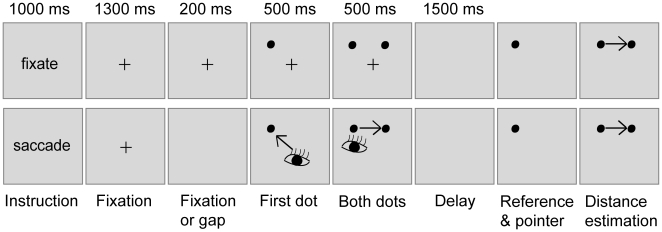
Consecutive stages of ‘fixate’ and ‘saccade’ trials. Horizontal separation between two sample dots had to be reproduced after a blank delay period. Distance estimation is based on a stationary reference dot and a movable mouse pointer that appeared on top of each other. The arrows with a depiction of an eye in the ‘saccade’ trial (lower panel) represent eye movements that brought the sample dots onto the fovea. The arrow in the distance estimation phase indicates a shift of the mouse pointer required to reproduce separation between the sample dots.

Two black dots having a diameter of 0.1 deg of visual angle served as the target stimuli and were displayed against a light-gray background. A pair of such dots was presented 5 deg of visual angle above the horizontal midline of the monitor at eight possible horizontal separations (from 2 to 16 every 2 deg of visual angle) (Figure 1). The horizontal position of a pair of sample dots was assigned on a trial basis by a randomly chosen, but predefined, shift to the left or to the right (from 2 to 3.5 deg of visual angle, every 0.5 deg) with respect to the vertical midline. The dots were not centered on the display in order to preclude subjects from using the vertical midline as additional positional information.

### Procedure

A single trial began with a 1000 ms presentation of instruction in the middle of the screen (Figure 1) that differentiated trials into two visual encoding conditions. If the word ‘fixate’ was displayed, the participants had to keep fixating at a subsequently presented central cross while the sample dots appeared 5 deg above on the monitor (Figure 1 upper panel). If the instruction read ‘saccade’, the subjects were required to move their eyes and foveate the sequentially appearing sample dots (Figure 1 lower panel). In ‘saccade’ trials a 200 ms blank gap was introduced in order to speed up a saccade toward the first presented dot [30]–[34]. The ‘fixation’ trials represented, therefore, registration of the stimuli by peripheral vision, whereas ‘saccade’ trials corresponded to stimulus encoding by foveal vision. The sample dots were presented sequentially in both encoding conditions with the first dot being displayed for 1000 ms, 500 ms individually and the last 500 ms simultaneously with the second dot. This presentation schedule disambiguated for ‘saccade’ trials the decision of which dot to foveate first. The leftward and rightward dots were equally often, but randomly, assigned as the first sample dot. During the sample dot presentation the central cross was displayed only in the ‘fixate’ trials while for the ‘saccade’ trials it was absent so as to aid fast eye movements toward the sample dots.

Presentation of the sample dots was followed by a 1500 ms blank interval - a delay period during which participants had to remember the distance between the sample dots. After the blank interval, a mouse cursor and one stationary reference dot were shown on the monitor. The cursor and the reference dots were similar to the sample dots. To prevent the initial cursor position from acting as a confounding spatial reference, it was always shown at the position of the dot that served as a reference for reproducing the distance. The reference dot together with the pointer reappeared in a different location than any of the sample dots. This reallocated reference position was calculated based on the position of one of the sample dots with a randomly chosen shift (from 2 to 3.5 deg every 0.5 deg) along the horizontal dimension either to the left or to the right. Irrespective of the visual encoding conditions, the subjects were instructed to use the mouse cursor to reproduce the sample distance in the horizontal dimension, based on the stationary reference dot, and then press the left mouse button when finished. After that response, the next trial began when the subject pressed the spacebar. Notably, observers were free to move their eyes and trace the mouse cursor during the distance reproduction phase in both, ‘saccade’ and ‘fixate’ trials that differed only in the stimuli encoding stage. Eventually, each of the sessions contained randomly interleaved trials varying in instructions with respect to the sample encoding conditions (peripheral - ‘fixate’ and foveal - ‘saccade’).

### Data analysis

The distance between the reference dot and the position of the cursor at the moment of the mouse-button press was calculated as the estimated distance. The estimation error (in deg of visual angle) was defined as the difference between the reproduced and the veridical distance. Statistical differences were assessed using a repeated-measure two-way ANOVA [35] with the sample encoding instruction (‘fixate’ and ‘saccade’) and the distance (eight spatial intervals between the sample dots from 2 to 16 deg) as main factors. We carried out the analysis on the absolute errors, signed estimation errors and standard deviation of absolute estimation errors. The first measure gives an overall accuracy, while the signed errors yield additional information with respect to a potential perceptual bias. Negative error values represent an underestimation of the distance, while positive values signify an overestimation of the sample distance. The standard deviation gives an indication of precision (variability) of responses.

In addition, the gaze position coordinates were calculated relative to the sample dot locations and the central cross with the aim to remove trials in which observers departed from instructions. We classified a ‘saccade’ trial as correct if the observer's gaze fell within a 2 deg - window around each of the sample dots of that trial. The ‘fixation’ trial was considered correct if the gaze stayed within a radius of 1 deg around the central cross during the sample dots presentation. Based on these gaze position criteria, on average, we collected 83% of total number of trials per subject (n = 7, *SEM* = 6%). From this eye tracker-filtered dataset we also left out trials with distance estimation errors larger than 3 *SD* of the grand average (in total 1.48% of trials). The further analysis of these ‘filtered’ trials shows that in ‘saccade’ conditions the observers on average initiated 2.7 saccades that were larger than 1 deg (SEM = 0.07) and 0.54 small saccades (from 0.3 deg to 1 deg in amplitude, SEM = 0.03). To follow the instruction and fixate both sample dots the subjects had to execute at least two saccades: from the fixation cross to the first sample dot and from there to the second dot.

### Modeling ‘repulsion effect’

Based on the estimated eccentricity dependent RF sizes we modeled the range and size of the repulsion effect as would be expected for the foveal and peripheral conditions. For the description of the population activity elicited by a sample dot we used a generalized Mexican hat distribution (second derivative of the Gaussian distribution) of the following form:
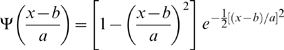
(1)


where *b* is the position parameter and *a* the dilation parameter. For our purposes parameter *b* represents the position of one of the dots (the position of the peak of the function). We assigned the position of the more foveal dot as zero and the location of the more peripheral of the two dots at the value of their separation, For our purposes, parameter *a* can be regarded as the radius of the average RF at a corresponding eccentricity. We based our calculations on the assumption that the mean RF size increases with eccentricity [36] and the two encoding conditions in our experiment while testing the same separations between the dots differed in the peripheral position of the more eccentric dot. We have no suppositions with regard to the likely visual brain area involved in our task, thus absolute RF sizes, and as a consequence this simulation can only be viewed as a qualitative description. A multitude of studies suggests a mathematical description of the linear relation between the RF size and eccentricity but they differ with respect to the measures used (e.g., a RF radius or diameter, classical RF with or without surround, perceptive vs. receptieve fields, with or without spatial attention, etc.) and the investigated visual area [37]–[42]. Eventually, we implemented the following rough but simple description of the scaling of the RF size in V1 with eccentricity [43], [44]:
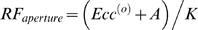
(2)


where *Ecc^(o)^* stands for the eccentricity in degrees of visual angle and *A* and *K* are constants (0.7 and 15, respectively). Since at the fovea the eccentricity should be near zero, we clipped the foveal RF size at 0.2282 deg [39], to avoid unreasonably small RF size ((0.7+0 deg)/15 = 0.0467 deg). With Equation 2 we estimated the relative RF sizes and thus the parameter *a* of Equation 1, at each eccentricity used in our study. Subsequently, we summed the Mexican hat functions representing each of the two dots for each separation and encoding condition and decoded the positions of the peaks relative to the superposition of these functions, which would reveal any perceptual biases.

## Results

We tested whether the visual encoding conditions, peripheral versus foveal vision, influenced accuracy in estimating a distance separating two co-planar dots. In the first condition the subjects had to keep fixating a central cross at the time of sample dots presentation that appeared 5 deg above the cross (‘fixate’ trials). In the second condition the observers executed saccades that brought the sample dots onto the fovea (‘saccade’ trials). The within-subject analyses of variance were carried out on the behavioral data from trials classified as correct (compliant with instructions) based on the eye movement recordings (see *Methods*).

**Figure 2:**
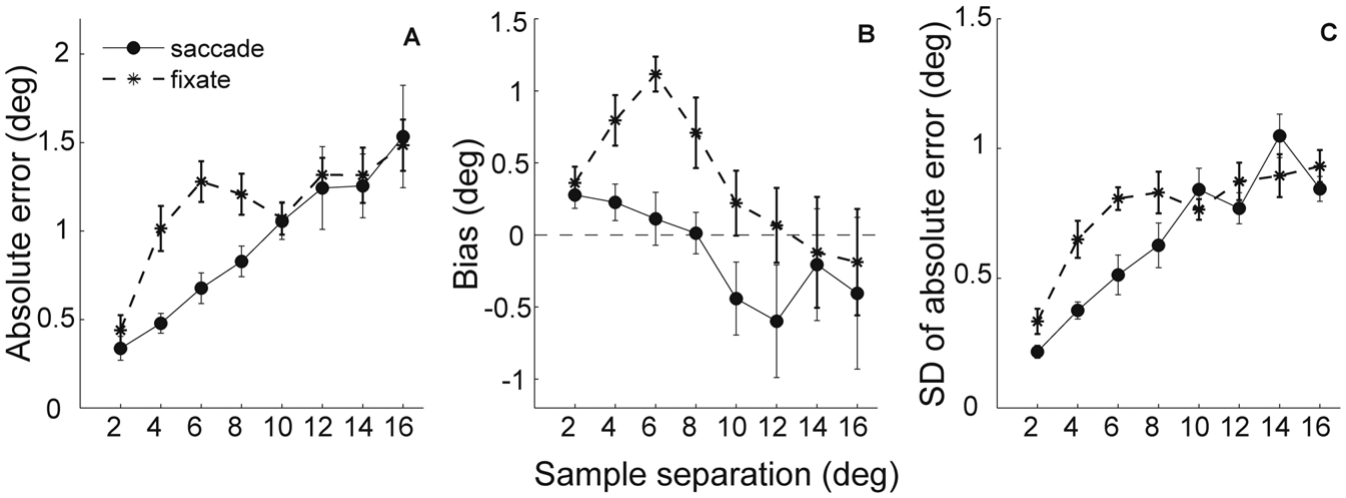
Estimation error as a function of sample distance. **A.** Absolute estimation error as a function of distance between two sample dots. The two encoding conditions: ‘saccade’ and ‘fixate’ (circles with a solid line and asterisks with a dashed line, respectively). **B.** Estimation bias as a function of sample distance. The sign of the estimation error indicates bias with negative values denoting an underestimation and positive values corresponding to an overestimation of the sample distance. Conventions are the same as in A. **C.** Standard deviation of the absolute estimation error as a function of separation between the sample dots. Conventions are the same as in A. Error bars denote *SEM* (n = 7).

Figure 2A shows the mean estimation error (n = 7) for the main conditions as a function of the sample dots horizontal separation. The pattern of absolute errors clearly indicates that the distance estimates were less accurate in the ‘fixate’ than in the ‘saccade’ trials (Figure 2A dashed line with asterisks and solid line with circles, respectively) and that was confirmed by the statistical analysis (encoding condition effect: F(1,6) = 7.69, p<0.05). From Figure 2A it is also apparent that the estimation error becomes larger with larger sample separations (distance effect: F(7,42) = 10.96, p<0.001). From the interaction of the two factors it becomes clear that encoding condition effect depended on the estimated distance with the performance in the ‘fixate’ trials being significantly worse only for distances 4, 6 and 8 deg (encoding condition and distance interaction: F(7,42) = 4.26, p<0.01; paired-sample t-tests for eight distances: p-value was less than Bonferroni corrected threshold p = 0.00625 only for distances 4, 6 and 8 deg).

In the following analyses we calculated estimation error that distinguishes over- and underestimations of the sample distance (bias in Figure 2B). Similarly to the absolute estimation errors, the encoding condition and distance as the main effects were significant and they interacted (encoding condition factor: F(1,6) = 22.73, p<0.01; effect of distance: F(7,42) = 4.80, p<0.01 and their interaction: F(7,42) = 2.99, p<0.05).

In general, subjects did not display any estimation bias if they foveated the sample dots during the encoding phase. Namely, t-tests demonstrated that the estimates in ‘saccade’ condition differed significantly from zero only for the smallest distance (t(6) = 3.0, p = 0.024). In the ‘fixation’ trials, however, the observers systematically overestimated the smaller sample dots' separations. To be more specific, p-values for the distances from 2 to 8 deg were less than 0.05 threshold (t(6) = 3.21, t(6) = 4.52, t(6) = 9.19 and t(6) = 2.90, respectively).

To get an indication of the consistency of the main effects of encoding condition and separation across subjects we carried out ANOVA's on individual subjects. In general, more than half of the participants (n = 7) showed significant effects of encoding condition and sample distance for both, absolute and bias errors. In particular, the encoding condition factor yielded p-values less than 0.05 in four subjects for the absolute estimation errors, and in five persons for the signed errors. The estimated distance factor modulated significantly performance in all observers when the absolute errors were considered and in four subjects for the bias measure. The interaction of the two factors was significant in two and three participants for absolute and signed errors, respectively.

The analysis of variance performed on the standard deviation of the absolute estimation errors tested the prediction that the two encoding conditions yield different scatter (variability) of responses. Figure 2C displays the standard deviation for the ‘saccade’ and ‘fixate’ trials as a function of the sample distance. It is apparent that the pattern of precision of responses resembles very closely the pattern of absolute errors. That is the two encoding conditions differed significantly (F(1,6) = 11.56, p<0.05) but interacted with the distance factor (distance effect: F(7,42) = 22.42, p<0.001, interaction: F(7,42) = 3.26, p<0.01). This similarity of the pattern of errors and its standard deviation reflect either the greater precision of responses in the ‘saccade’ condition or simply the natural relation of the smaller deviation with the smaller values of error.

**Figure 3:**
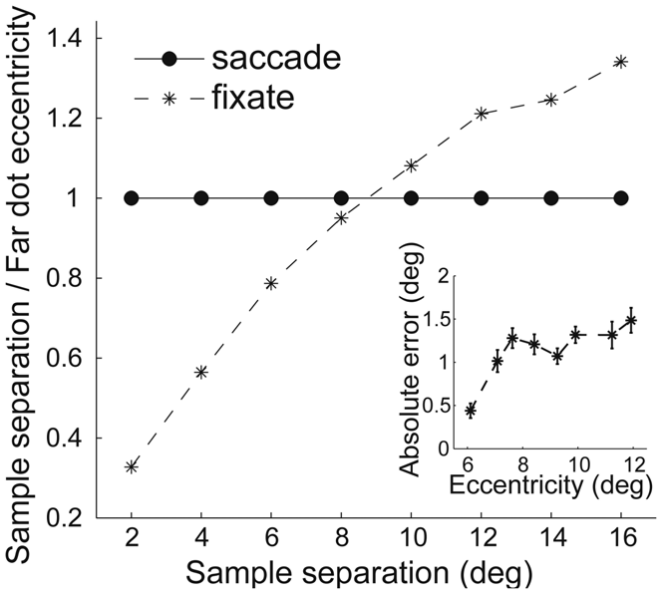
The ratio of separation by eccentricity as a function of that separation. For the ‘saccade’ (solid line with circles) condition, the eccentricity corresponded directly with the distance between the sample dots. For the ‘fixate’ (dashed line with asterisks) encoding condition, the eccentricity was calculated as the mean eccentricity of the most peripheral dot of a sample pair of dots.

In the light of eccentricity-dependent positional uncertainty, it is of importance to know the average eccentricity of the most peripheral sample dots, which would limit the precision of the localization task [45]. In the ‘saccade’ condition while foveating one of the sample dots the horizontal separation between the dots corresponded directly with eccentricity. Contrary to that, in the ‘fixate’ condition, the eccentricity of the most peripheral dot differed from the sample distance. We calculated the mean eccentricity of the peripheral dot and plotted it as a fraction of sample separation. Figure 3 illustrates the difference between the foveal and peripheral encoding conditions with respect to the furthest dot eccentricity and the separation between the dots. The separation by eccentricity ratio in ‘fixate’ trials changes from less than 1 to larger than 1 as a function of the presented distance, which is in contrast to the constant ratio of 1 in ‘saccade’ trials.

**Figure 4:**
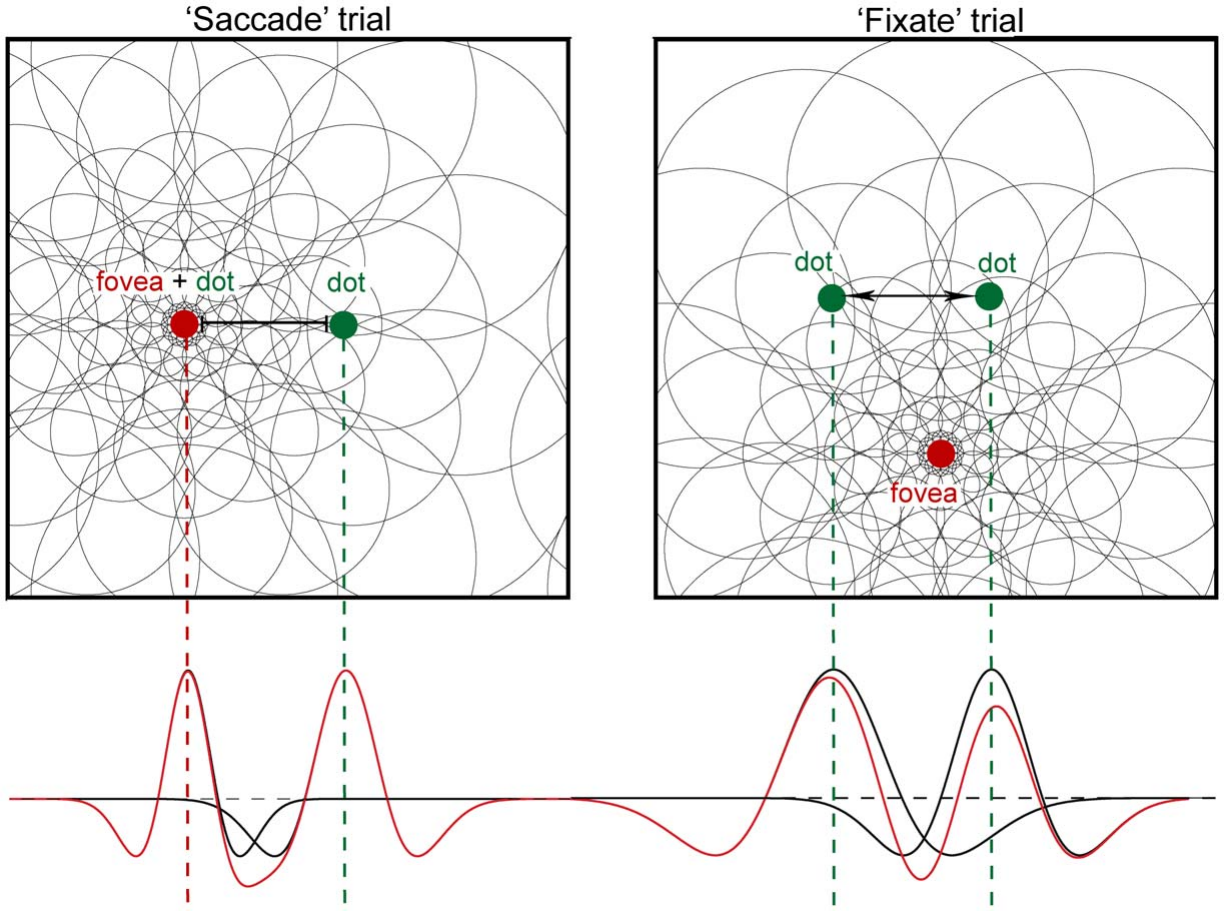
Theoretical neuronal populations response to a pair of dots. The green circles illustrate two sample dots presented on a monitor. The red circles represent gaze position relative to the sample dots. The left panel represents a ‘saccade’ trial when an observer foveates the leftward dot and at the same time encodes the rightward dot by peripheral vision. The right panel represents a ‘fixation’ trial when an observer foveates a central cross while both sample dots are encoded by peripheral vision. The empty circles of variable size correspond to receptive fields (RFs) covering visual field. The lower panels characterize neuronal populations responses to the visual stimuli in the ‘saccade’ (left) and the ‘fixate’ (right) conditions. The peaks of responses correspond to the positions of the dots when viewed individually (black lines) and when viewed simultaneously (red lines, representing the sum of the functions in black). The line between the sample dots in the ‘saccade’ condition symbolizes no direct interactions between the neuronal populations (small overlap of RFs). The arrow between the sample dots in the ‘fixation’ condition signifies a repulsive effect in perceived distance (large overlap of RFs).

**Figure 5:**
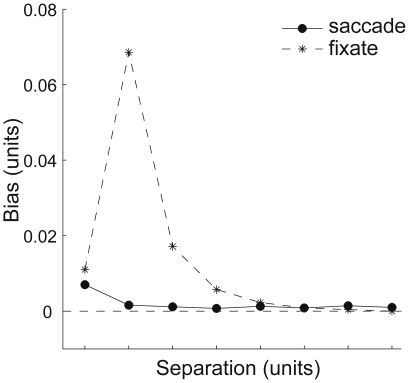
Bias in distance estimation based on the RF sizes predictions. The positive values represent an overestimation of a separation between two dots and the negative values, an underestimation. The difference in the eccentricity-related size of the RFs taxed by the ‘saccade’ (solid line with circles) and ‘fixate’ (dashed line with asterisks) conditions, results in a divergent pattern of distance estimation bias.

We further modeled the consequences of these differences in separation by eccentricity ratio in the context of the repulsion effect. Based on the decoded positions of the peaks of the summed distributions of the populations' activity (red lines in Figure 4) we obtained qualitative predictions of the range and size of the repulsion effect for the foveal and peripheral conditions. The difference in decoded locations (separations) for the ‘fixate’ and ‘saccade’ trials are plotted in Figure 5, with the positive values representing a greater distance between the peaks in the summed function than in the superposition of these functions, an overestimation bias. Considering these simulations one would therefore expect a more substantial repulsion effect in the peripheral encoding condition than in the foveal trials, which is indeed apparent in observers’ distance estimates (compare Figures 2B and 5).

## Discussion

The current experiment was designed to clarify whether estimation of a distance between two objects (dots) in the frontoparallel plane is influenced by the way in which the visual-spatial information is acquired. For that purpose we manipulated the instructions to either ‘fixate’ a central cross during the sample presentation or to ‘saccade’ towards the appearing sample dots. In the first case, visual information is obtained via peripheral vision while in the second case the sample dots are foveated and additionally encoded by central vision.

The main finding is unambiguous: fixating the sample dots improves the subsequent reproduction of a distance separating two sample dots in both, general accuracy and in precision of responses. Interestingly, the beneficiary effect of fixating the targets is limited to the smaller distances, up to 8 deg. For larger separations between the two dots the reproduction errors and their standard deviation do not differ between ‘fixation’ and ‘saccade’ conditions.

It is very useful to consider our experimental design and findings in the light of eccentricity-dependent spatial uncertainty and Weber's Law. On the one hand, the first phenomenon has its origin in the anatomy and physiology of the retina and cortex that results in a sparse neural sampling grain of the peripheral visual field [46]. On the other hand, Weber's Law in the context of our study predicts that the position threshold is approximately proportional to the separation. The applicability of such a linear relation between the localization performance and the separation of the reference features has been found only for stimulus configurations where the ratio of separation by eccentricity is higher than 0.5 [45]–[47]. At these separations the eccentricity of the furthest stimulus would become a limiting factor of the localization performance. If we examine Figure 3 it becomes apparent that the foveal and peripheral encoding conditions in our experiment differed greatly with respect to the separation by eccentricity ratio. To be exact, in the ‘saccade’ trials, when the observer's gaze is fixed on the first sample dot, the second one is registered by peripheral vision at the eccentricity directly corresponding with the sample distance (Figure 3, solid line with circles). In the ‘fixate’ trials, however, there is no 1∶1 relation between sample dots' eccentricity and the separation between them. The smaller distances corresponded with a larger eccentricity and the distances larger than 10 deg were presented at smaller eccentricity than in the ‘saccade’ trials. Ultimately, only the smallest separation in the ‘fixate’ condition was within the regime of the Weber's Law (ratio lower than 0.5), whereas the remaining conditions should be mainly influenced by the eccentricity of the furthest dot. While the absolute errors in the foveal conditions show an approximately linear relation with separation/eccentricity (Figure 2A) the peripheral condition does not appear to be similarly affected by eccentricity (insert in Figure 3). The pattern of results, therefore, cannot be simply explained in terms of eccentricity-dependent positional uncertainty.

Alternatively, instead of considering our results as an improvement of distance estimates due to foveating the visual targets, one can frame it as a reduction in a bias that emerges when both closely spaced targets are viewed peripherally. When we take into account the signed error measure of performance, it becomes clear that the subjects systematically overestimated smaller distances in the ‘fixation’ conditions. This pattern of responses brings to mind the repulsion phenomenon in motion direction perception [14], [15], orientation discrimination [16]–[19], and stereoscopic depth perception [20], [21]. In short, observers tend to perceive small differences in orientation, direction or depth as being larger, which is not observed for larger dissimilarities. For instance, in a study on perception of motion direction, Rauber & Treue [48] used a control experiment, in which the subjects had to judge spatial separation between a reference line and the centre of a circle. Similar to the results of motion direction judgments, the researchers found the smaller separations between the line and a circle to be overestimated and suggested that such repulsion is a general phenomenon [48], [49]. Accordingly, many researchers postulate that repulsion effect in motion direction perception, orientation discrimination, and depth perception are a direct consequence of physiological organization of receptive fields due to e.g., centre-surround and lateral interactions [18], [19], [21] (for a review, see [50]). To give an example, it is known that both simple and complex cells display spatial segregation of excitatory and inhibitory interaction within their RF that can be even opposing depending on the spatial context of the target stimulus [51], [52].

Crucially, the results of our model simulations bear the notion that the distance estimation bias we recount here reflects the repulsion effect reported for other discrimination tasks. Although the relative overlap of receptive fields is independent of eccentricity [53], the spatial range of interactions is greater for larger RFs and hence eccentricities. In the ‘fixation’ condition the sample dots were shown parafoveally/peripherally. When they appeared at small separations the resulting neuronal activity coding for the location of each of the two dots was overlapping which consequently induced inhibitory interactions between the two populations of cells and an overestimation of remembered distance (Figure 4, upper right panel). Since the spatial range of neuronal interactions is limited, one observes the repulsion effect only for smaller distances. On the other hand, in the ‘saccade’ condition when one of the dots is foveated the other is encoded by the parafoveal/peripheral vision (Figure 4, upper left panel). Thus, the retinal error is calculated from the viewing point of either of the stimuli. Because central vision relies on smaller RFs than the peripheral vision, one would expect less overlap in neuronal activity elicited by the two sample dots in this condition and accordingly, less neuronal inhibition (Figure 4, compare lower left and lower right panels). This proposal can be supported by the fact that for the smallest separation (2 deg) the subjects overestimated distance in ‘fixation’ and ‘saccade’ conditions to the same extent while distances between 4 and 8 deg were overestimated only in the ‘fixation’ trials. In order to verify this notion we designed a model, of which details can be found in the *Methods* section. We have to stress that it is not a quantitative model but a qualitative description of how the pattern of results we found might be explained by the differences in the RFs sizes involved in the two encoding conditions. In short, we used a classic Mexican hat distribution to describe the neuronal activation pools elicited by presentation of the two sample dots. The width of the distribution reflected the estimated RFs width at the particular eccentricity, corresponding to those used in the experiment. To calculate these widths we used the relative differences in stimuli eccentricity implemented in the ‘saccade’ and ‘fixate’ trials (it can be also inferred from Figure 3) and the positive linear relation between mean receptive field size and eccentricity [36] (see section *Modeling ‘repulsion effect’* for the equations and points of consideration). Subsequently, we varied the separation between the two peaks of the activation pools representing distances between the sample dots. In a single stimulus condition the decoded position of the dot was calculated as the position of the peak of activity. When the two dots are presented simultaneously, the resulting positional decoding would correspond with the peaks of the summed distributions (see Figure 4, red lines in the lower panels). When there is no direct interaction due to a large separation and/or small RF sizes, the summed distribution becomes a superposition of the activity elicited by two individual dots (Figure 4, lower left panel). When the widths of the RFs are large enough and the separation between the two stimuli small enough, the interaction between the neuronal populations results in an outward shift of the peaks of the summed distributions (Figure 4, lower right panel). Crucially, the separation in the two encoding conditions was kept the same and only the eccentricity-related RF sizes differed between foveal and peripheral encoding. Figure 5 depicts the ‘saccade’ and ‘fixate’ trials for eight equally spaced stimulus distances and the corresponding biases calculated from the positional shifts of the peaks of the summed distributions. The comparison of Figure 5 with Figure 2B leaves little doubt that the proposed mechanism very likely influenced the pattern of distance estimation errors in our experiment.

Even though the current results fit very nicley the framework of the repulsion effect, we cannot exclude the possibility that the distance estimates in our experiment were to some degree influenced by the oculomotor signals associated with saccades bringing the two sample dots onto the fovea. To be explicit, the information about the gaze direction at the moment of foveating the sample dots and the amplitude of the saccade spanning the two dots could enhance the performance in the ‘saccade’ condition relative to the ‘fixate’ trials. Relevantly, such improvement would be especially pronounced in the situation when the pattern of oculomotor behavior is re-evoked during the distance reproduction phase. However, in the current design the reference dot was displayed at a different location than any of the sample dots yielding the gaze direction signal less informative. With respect to the saccade amplitude effect, on average subjects performed at least one corrective saccade during the sample presentation, which questions the usefulness of the saccade amplitude information. More importantly, during the retrieval phase the subjects fixated the reference dot only in about 65% of the ‘saccade’ trials thereby diminishing the possible usage and influence of oculomotor signal in the foveal encoding condition. Taken these issues into account we believe that if the oculomotor activity indeed contributed to the observers' performance in our experiment, this influence was relatively insignificant in comparison with the effects of encoding conditions per se.

To sum up, we demonstrate that encoding and retrieval of a distance separating two items is improved by foveating the sample dots. Although, we cannot definitely exclude the reduction of the eccentricity-dependent positional uncertainty as a factor contributing to some degree in such accuracy increase, the presence of the systematic overestimation bias points to other sources influencing performance. We favour the notion that the foveal encoding reduces a perceptual bias that emerges when the stimuli are presented more peripherally. The foveal distance encoding condition assures that the stimulated neuronal populations do not overlap and thereby the inhibitory interactions supposedly underlying perceptual repulsion are precluded. For larger distances the way of encoding, peripherally or foveally, does not influence distance estimation in our experiment.
